# Prediction of Individual Dynamic Thermal Sensation in Subway Commute Using Smart Face Mask

**DOI:** 10.3390/bios12121093

**Published:** 2022-11-29

**Authors:** Md Hasib Fakir, Seong Eun Yoon, Abdul Mohizin, Jung Kyung Kim

**Affiliations:** 1 Department of Integrative Biomedical Science and Engineering, Graduate School, Kookmin University, Seoul 02707, Republic of Korea; 2 Department of Mechanical Engineering, Graduate School, Kookmin University, Seoul 02707, Republic of Korea; 3 School of Mechanical Engineering, Kookmin University, Seoul 02707, Republic of Korea

**Keywords:** wearable biosensors, smart face mask, skin temperature, exhaled breath temperature, thermal sensation vote, machine learning

## Abstract

Wearable sensors and machine learning algorithms are widely used for predicting an individual’s thermal sensation. However, most of the studies are limited to controlled laboratory experiments with inconvenient wearable sensors without considering the dynamic behavior of ambient conditions. In this study, we focused on predicting individual dynamic thermal sensation based on physiological and psychological data. We designed a smart face mask that can measure skin temperature (SKT) and exhaled breath temperature (EBT) and is powered by a rechargeable battery. Real-time human experiments were performed in a subway cabin with twenty male students under natural conditions. The data were collected using a smartphone application, and we created features using the wavelet decomposition technique. The bagged tree algorithm was selected to train the individual model, which showed an overall accuracy and *f*-1 score of 98.14% and 96.33%, respectively. An individual’s thermal sensation was significantly correlated with SKT, EBT, and associated features.

## 1. Introduction

Urban traffic is becoming a prominent problem with the development of urbanization [1]. While public transport on the ground is facing huge pressures in many cities due to the increasing number of passengers and vehicles, the subway is becoming popular in metropolitans, such as Seoul. The underground railway network has become an integral part of the public transport system because of its high speed, large passenger capacity, and timely operation. With the rising demand for the subway and its influence on public health, the subway’s thermal environment has also attracted the attention of researchers [2]. Therefore, it is necessary to evaluate the thermal comfort of the underground train carriage to ensure a healthy and comfortable thermal environment [3], which will also facilitate determining an energy management scheme to minimize the energy consumption of the subway [4]. Typically built environments consist of indoor, outdoor, and transitional spaces [5]; however, the subway-built environment differs with high mobility, short passenger stay times, and high passenger density. Hence, thermal comfort in the subway needs to be dynamically considered using an individualized prediction model.

Previous studies have been extensively conducted on the thermal comfort of indoor and outdoor environments based on the climate chamber, field study, and simulation methods [6,7,8,9]. Previous investigations included different climatic zones [10,11,12] and were mainly focused on various built environments, such as offices [13], residential buildings [14,15,16], educational institutes [17], automobiles [18,19,20,21,22], and museums [23,24]. They considered both physiological and ambient parameters, including skin temperature, heart rate, ambient temperature, air velocity, relative humidity, and globe air temperature, to predict a thermal sensation for specific built environments. Many of these studies used environmental sensors, thermal imaging [25,26], and wearable sensors to measure heart rate [27,28,29] and skin temperature [30,31,32,33] from different body parts such as the forehead, wrist, leg, thigh, and back during the experiments. These studies were conducted in climate chambers with controlled environmental conditions and can be a close approximation of certain realistic conditions. However, they are a poor approximation of dynamic environments, such as subway commutes, where a significant percentage of the population uses the environment on a daily basis.

Thermal comfort studies on subways have received a lot of attention in recent years with the increasing number of underground railroads. Han et al. [34] conducted a study on the subway of Seoul during three different seasons and found a significant difference in air temperature during the different seasons. The study considered thermal, air, acoustics, light, and overall comfort. Although most respondents felt neutral or comfortable, this study did not identify thermal comfort at an individual level. The hypothalamus in the brain controls thermoregulatory mechanisms, and thermal sensations can be predicted from brain activity signals. The power of the beta and gamma bands significantly increased in an uncomfortable subway station during the experimental study by Kim et al. [35]. They chose one uncomfortable and one comfortable subway station, and by using an electroencephalogram (EEG) device, the participant’s brain activities were recorded in a sitting position. However, due to the complexity of wearing an EEG device and the motion interference, it was not convenient to use an EEG signal for predicting thermal sensations while using the subway.

A study by Abbaspour et al., which investigated thermal sensations in Tehran subway stations and train carriages, found that the relative humidity was low in the Tehran subway [36]. They also found that the temperatures in the entrances, station halls, and platforms were higher than the temperature of the train. The depth and design of the subway stations affected the thermal condition, as found by a study in Athens [37]. Zhang et al. [38] pointed out the factors of the tunnel’s thermal environment in China. Train density and passenger flow have the most important impact on the subway tunnel’s thermal environment. Yang et al. [39] conducted a long-term field study in the cold region of China, Harbin. They calculated the minimum comfortable temperature for the aisle, hall, and platform of the subway station and found that 70% of the passengers felt comfortable with the existing thermal environment. However, this study did not investigate thermal comfort in train cabins. All the studies above have contributed to the literature on the subway’s thermal environments with some limitations, such as measuring from static points, using a population model, and using a complex sensor setup. Passengers go through various points while using a subway train, including the platform, train cabin, and entry and exit points. Passengers’ activities also vary depending on the situation, such as waiting on the platform, transferring to trains, and sitting or standing during travel, which indicates the necessity of a dynamic thermal sensation prediction model.

In this study, a naturalistic investigation was performed with the aim of making a smart face mask to predict an individual’s thermal sensation in a dynamic environment, such as a subway commute. In our previous study, we reported the feasibility of using a smart face mask to obtain an individual’s comfort state [40]. Physiological and psychological responses of subway passengers were measured. Only biological signals that could be obtained from the sensors embedded in the smart face mask, such as skin temperature (SKT) and exhaled breath temperature (EBT), were recorded, and the corresponding thermal sensation votes (TSVs) were collected every 5 min using a mobile application. The transient EBT infers crucial biological information, including core body temperature and metabolism, and can be utilized as an effective quantifier of the body condition. In the present study, these biological signals and corresponding TSVs were utilized to obtain the personalized thermal comfort model, which achieved an overall accuracy of 98.14%.

## 2. Materials and Methods

Herein, the thermal sensation of passengers during a subway commute was investigated with the aid of a developed smart face mask. The transient EBT and SKT signals and corresponding TSV were recorded with a developed smartphone application. The study aimed to investigate the thermal sensation characteristics in a real-world scenario and hence it was designed to have minimal deviation in a passenger’s routine. We also applied a machine learning algorithm to obtain a personalized thermal sensation model with the measured data.

### 2.1. Smart Face Mask

#### 2.1.1. Sensors

Since the experiment was chosen to be a naturalistic experiment where the participants could mingle with other people in their daily routines, appropriate sensor selection was a critical factor. The appropriate sensor would not cause any inconvenience to the participants and the passengers around them. Thus, certain conditions were to be followed:

- It should be as non-invasive as possible.
- Analog data should be easily accessible.
- It should be as comfortable as possible for use in daily life and should be portable.

**Figure 1:**
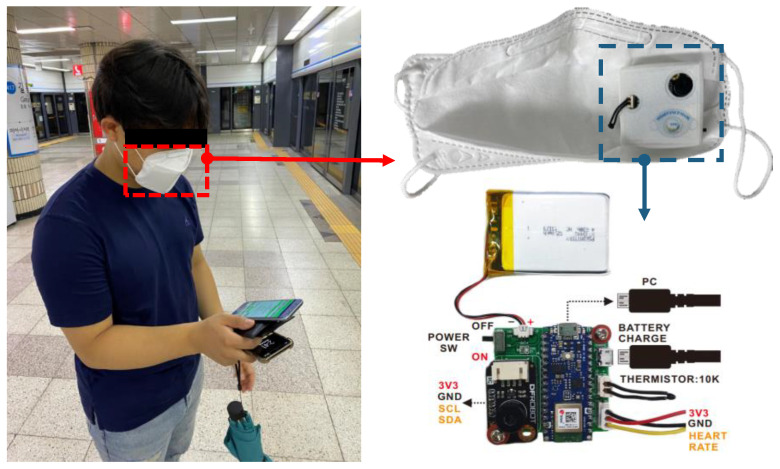
Details of the developed smart face mask with the embedded sensors.

For this study, we measured SKT by a non-invasive infrared temperature sensor (MLX90614, Melexix Technologies NV, Tessenderlo, Belgium) that detects infrared rays emitted from an object through a built-in heat medium and converts them into an electrical signal [41]. EBT was measured by a thermistor (NXFT15XH103FA2B130, Murata Manufacturing, Kyoto, Japan), which is a negative temperature coefficient sensor with a resistance of 10 kΩ at 25 °C. Sensor information is provided in Table 1. All sensors were controlled using an Arduino (Arduino NANO BLE, Sparkfun Electronics, Niwot, CO, USA) and placed inside the mask (Figure 1).

#### 2.1.2. Mobile Application Development

**Figure 2:**
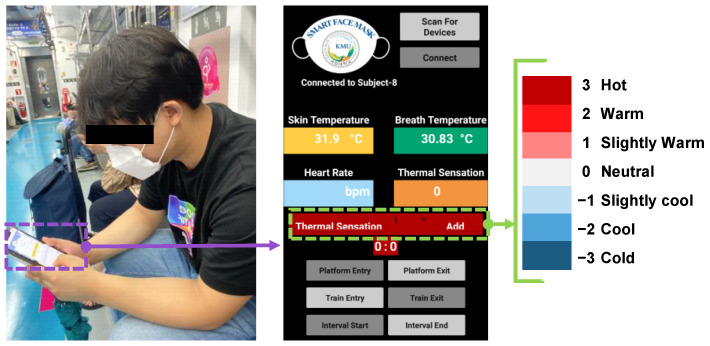
Mobile interface for thermal sensation votes (TSVs), skin temperature (SKT), and exhaled breath temperature (EBT).

Physiological data measured by the smart mask were monitored by an android mobile application, and the layout is shown in Figure 2. The application was developed using the MIT app inventor, initially provided by Google and is currently managed by MIT [42]. Initially, the user is asked to initiate the connection with the device using the required button after wearing the smart face mask (Video S1). If the connection is successful, the status in the text box below the scan button will change to connected. The application can show the current SKT and EBT, and there is a dropdown menu to the right of the thermal sensation box to select the user’s TSV, which they can save by using the add button. Just below this, there is a timer that shows the time after submitting each vote and will also give an alarm at the end of 5 min, so the user does not miss any voting (Video S2).

### 2.2. Participants Selection

Twenty participants, including Korean and foreign nationalities (11 and 9 participants, respectively) who have lived in Seoul for more than 1 year, were chosen for this study based on physiological and health status surveys. Table 2 gives the average age and physical information of the participants. The experimental procedure was explained in detail by pretraining prior to the experiments for a better understanding of the experiments. The participants were asked to sign a consent form before participating in the experiments. The participants were asked to avoid caffeine, alcohol, and extreme physical work for 12 h prior to the experiments. The participants wore short-sleeved T-shirts and long pants based on the summer/autumn clothes of a university student.

### 2.3. Experimental Procedure

**Figure 3:**
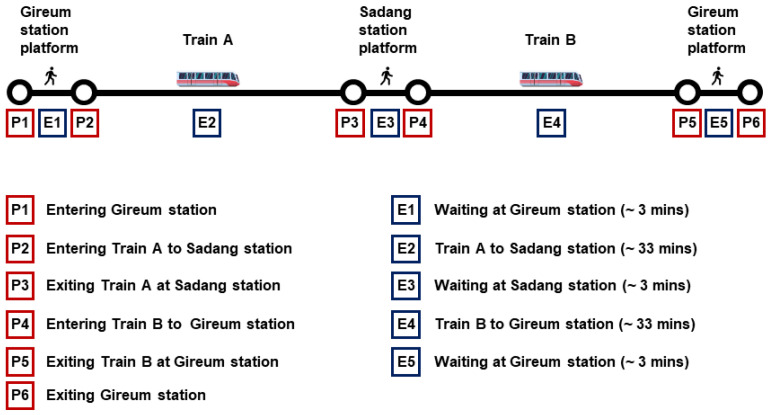
Schematic representation of the naturalistic experiment done, divided into events.

The goal of this research was to predict thermal sensation outside the laboratory setting where people were performing their regular life activities. The participants wore the smart face mask and traveled on the Seoul metropolitan subway line number 4 from Gireum to the Sadang station and returned in the same way as shown in Figure 3. The experiments were performed between 23 August and 27 August 2021. The average outdoor temperature and relative humidity were 23.74 °C and 87%, respectively. August is traditionally one of the hottest months in Seoul, with high relative humidity and heavy rainfall. Hence, a subway cooling system is usually employed to generate a comfortable thermal environment for the passengers. The duration of each experiment was about 70 min and was repeated on 3 different days during the same time. The study was not conducted during the rush hour and the information regarding the average number of people during the commute is provided in Table A1. The participants were not accompanied and commuted normally except for entering the TSVs during their travel. As the study was a naturalistic investigation, no control was made to the environment or to the participants’ metabolic activities (sitting or standing) and their time waiting for the train.

### 2.4. Data Collection and Analysis

SKT, EBT, and TSVs were collected from the participants using the smart mask and mobile application. SKT was measured from the cheek of the participant’s face, and the air temperature inside the face mask was recorded for the EBT. Data were transferred to the mobile application through Bluetooth and saved to the phone’s local storage. Participants gave their TSVs on a 7-point scale using the application every 5 min. All the physiological data were collected with a 1 Hz sampling rate. To increase the data volume for use in machine learning, each participant took part in this experiment 3 times but not more than once a day. Data collection started when the participant entered the station by pressing the platform entry button and finished when the participant exited the station by pressing the platform exit button. Finally, the participant sent the stored file by email after finishing each experiment.

**Figure 4:**
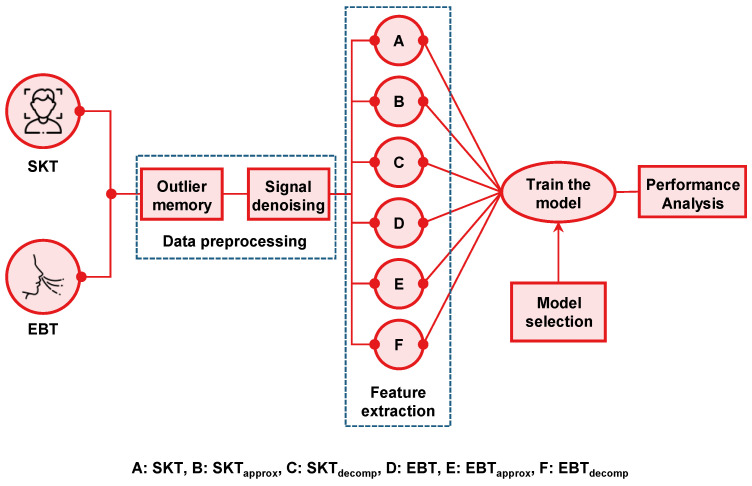
Overall block diagram for making individual thermal sensation prediction model.

**Figure 5:**
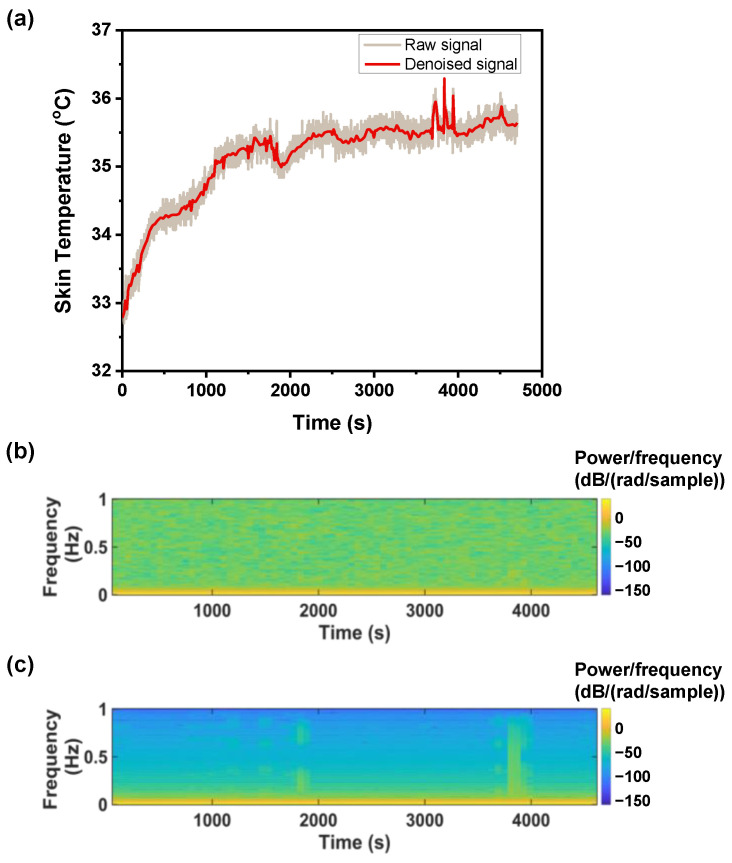
Extracting skin temperature: (**a**) recorded skin temperature with noise reduction, (**b**) spectrogram for recorded skin temperature, and (**c**) spectrogram for the denoised skin temperature.

The overall data analysis procedures are shown in Figure 4. Before applying the machine learning algorithm to predict the individual thermal sensation of subway passengers, data were preprocessed to remove outliers (greater than 3 standard deviations) and null values. Participant 6′s data were not recorded correctly and were excluded from the analysis. Participants 1, 8, 14, and 17 had difficulty one day acquiring data. Hence out of the 60 cases recorded from the 20 participants, 53 data samples were suitable for analysis. The recorded SKT signal is shown in Figure 5a with a brown line. The SKT and EBT signals were highly contaminated with artifacts because of the ambient temperature and airflow during talking and walking. These high-frequency signals were possibly dominant, making it difficult to extract the original signal in the time domain, as shown in Figure 5b. We applied the wavelet denoising technique to approximate the original signal without losing any temporal information, as shown by the red line in Figure 5a. We made a spectrogram to observe the temporal information using short-term Fourier transform to represent the data in the time–frequency domain. The spectrogram was made using the Hann function, and the window size was selected for 3 min with 2 min overlapping.

**Figure A1:**
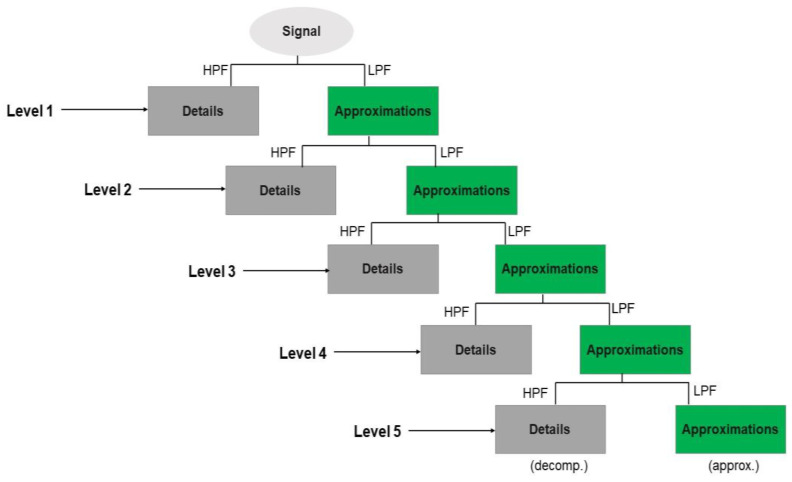
Feature creation using wavelet decomposition.

Figure 5c shows that after removing the noise, the signal has consistent high energy in the lower frequency ranges. The lower frequency bands were extracted using the wavelet-based decomposition technique and were made into features for machine learning. Wavelet decomposition is a time–frequency analysis that provides information in both the time and frequency domains of a signal and is widely used for biomedical signal processing [43,44,45,46]. Wavelet decomposition is a bandpass in nature and divides the frequency spectrum into two halves of equal frequencies. The lower frequency band is further subdivided into two equal halves (Figure A1). The selection of the mother wavelet and the number of the approximation level is important for the wavelet signal decomposition. Daubechies was selected as the mother wavelet for the ideal natural signal, as suggested by Rafiee et al. [43], and the decomposition was performed up to level 5. The low- and high-frequency band signals from the tertiary level were selected as features along with the denoised SKT and EBT. Hence, a total of six features were selected for further analysis with machine learning: SKT, SKT_approx_, SKT_decomp_, EBT, EBT_aprrox_, and EBT_decomp_. The pseudo-code for feature creation is given in Algorithm 1.

**Algorithm 1:** Pseudo-code for wavelet-based feature creation
**Algorithm To Construct Features Vector**
**Input:** recorded SKT, EBT, and TSVs **Output:** table with two more features for each signal, and TSVs **Begin:**   read data table;   **FOR** each SKT:       **IF** (SKT is outlier **OR** null):       remove the row; **       END IF**;   **END FOR**;   **FOR** each EBT:       **IF** (EBT is outlier **OR** null):       remove the row;       **END IF**;   **END FOR**;    **denoise** SKT and EBT using MATLAB function;   **decompose** SKT and EBT using wavelet decomposition function;   **reconstruct** SKT approximation and details;   **reconstruct** EBT approximation and details;   **make table** with SKT, EBT, associated approximation and details, and TSV; **END**;

## 3. Results and Discussion

Thermal sensation prediction in various environments is an extensively studied area. Various static and dynamic models were proposed for particular environments based on the local sensation parameters of the human body [47,48,49,50]. However, obtaining temperature profiles of various body parts with wearable sensors is not yet practical for the normal daily routine of a person. Using a smartwatch as a sensor could achieve biological signals in real-time and is being explored for various purposes in health and wellbeing [51]. Due to the impact of COVID-19, face masks have become an integral part of people’s day-to-day lives and provide exhaled breath data, which provides unique information in real-time during day-to-day activities and would not have been possible before the COVID-19 pandemic. Various applications using the exhaled breath data have been reported recently [52,53,54,55], and in our previous work [40], we proved the feasibility of using such a system for analyzing the thermal comfort of a person. In the present study, a smart face mask was developed and coupled with a mobile application to investigate the thermal sensation in a public subway.

### 3.1. Analysis of Physiological and Psychological Datasets during Subway Commute

**Figure 6:**
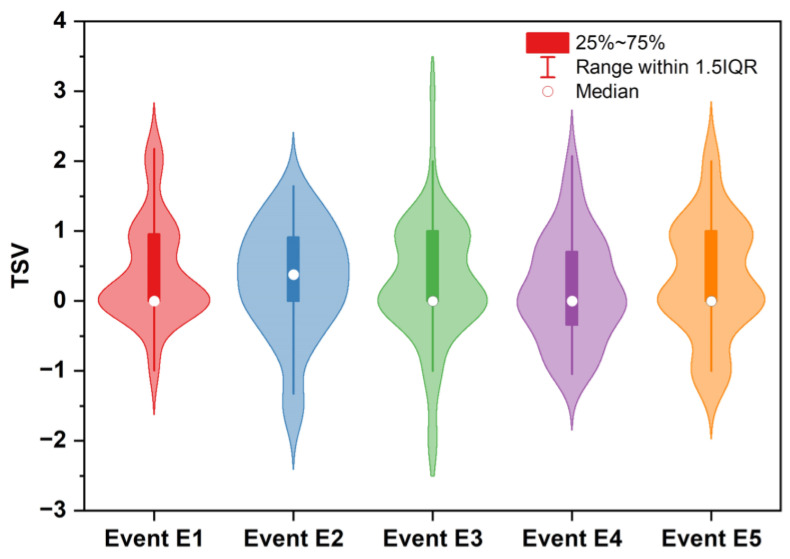
Average recorded TSVs for each event. *n* = 53 cases.

The average TSV for the 53 cases is shown in Figure 6. The majority of TSVs were between 0 and 0.5 for all events (Figure 6). Thus, about 50% of the subjects felt a neutral thermal sensation, and if a linear relation for the preference of comfort is assumed, they were in the comfortable range throughout the commute. However, the plot shows that the data distribution is multi-modular, which suggests that the subpopulation of the dataset that was on the platform is distinct from that inside the subway cabin.

**Figure 7:**
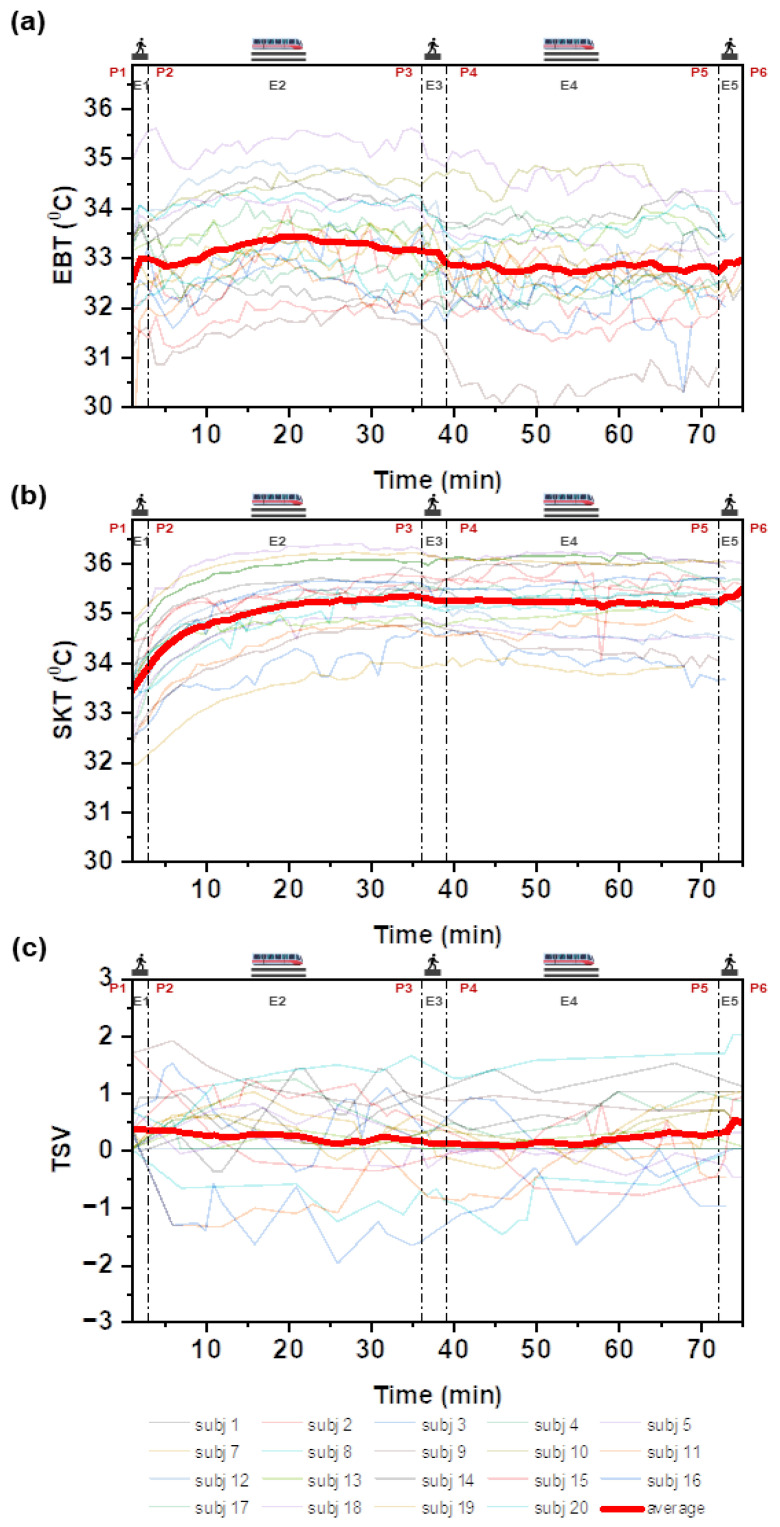
Average transient (**a**) EBT, (**b**) SKT, and (**c**) TSVs for each participant.

The average transient variation of SKT, EBT, and TSVs of the participants along the duration of the experiment is shown in Figure 7. EBT is sinusoidal in nature and increases during exhalation and decreases during inhalation. The average EBT and SKT in the transition period from P5 to P6 increased because of their previous thermal experiences and were more controlled in the closed train cabin than in the semi-controlled platform or exit. This observation was reinforced by their TSVs during P5 to P6, as shown in Figure 7c. The average TSV increased while leaving the train and heading toward the platform and exit, which may have affected the increased EBT.

**Figure A2:**
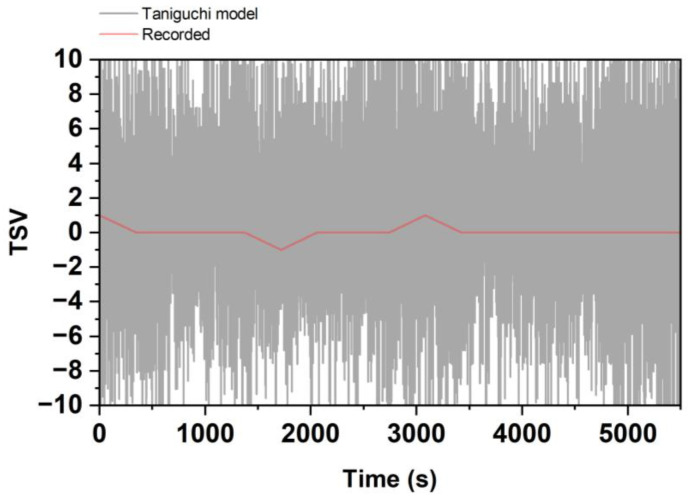
Fitted Taniguchi model [56] for subject 1, case 3.

There are limited models which have studied asymmetrical environments and transient conditions since they are more complex than uniform environmental conditions because people’s responses depend on the comfort of their local body parts and that of their whole-body comfort. Thus, the correlations developed from such scenarios include the response of various body parts and could not be applied to this study. However, in a study by Taniguchi et al., a multi-linear regression model was used to correlate TSVs with the transient facial skin temperature and its rate of change in a vehicle environment [56]. However, the correlation could not predict TSVs in the present study since it only focused on the facial skin temperature variation (Figure A2). Since other traditional TSV prediction models require local sensation data [47,48,49,50], they could not be applied to this study; hence an AI model was used to obtain a personalized thermal comfort model.

### 3.2. Personalized Thermal Comfort Model

This study aimed to predict individual TSVs based on the physiological parameters. Participants gave their TSV on a 7-point thermal sensation scale ranging from a score of −3 to +3, and a classification model was made for each participant. Since the sampling rate of the temperatures and TSVs was different, the TSVs were interpolated using a linear approximation model to obtain the transient TSV profile, and its relationship with the other input features was estimated using their correlation, as shown in Table 3. Correlation is used to measure the association between two variables and quantified by the correlation coefficient. The correlation coefficient varies between −1 and 1, which represent no relation and strong linear relation between the variables, respectively. The input features showed significant (*p* < 0.01) correlations with low to moderate correlation coefficients. It can be inferred that SKT, EBT, and the associated features increase with increasing TSVs, but not all the features increase at the same time. Moreover, individuals have different strong predictive features associated with their TSVs. For example, the EBT is more correlated with TSVs for participant 1, but SKT is more correlated with TSVs than other features for participant 7. We can identify which features to use and reduce the number of features based on this correlation matrix. Among the participants, participant 12 showed no change in their TSVs for all 3 experimental days, so participant 12′s data were excluded from the individual TSV prediction model.

**Figure 8:**
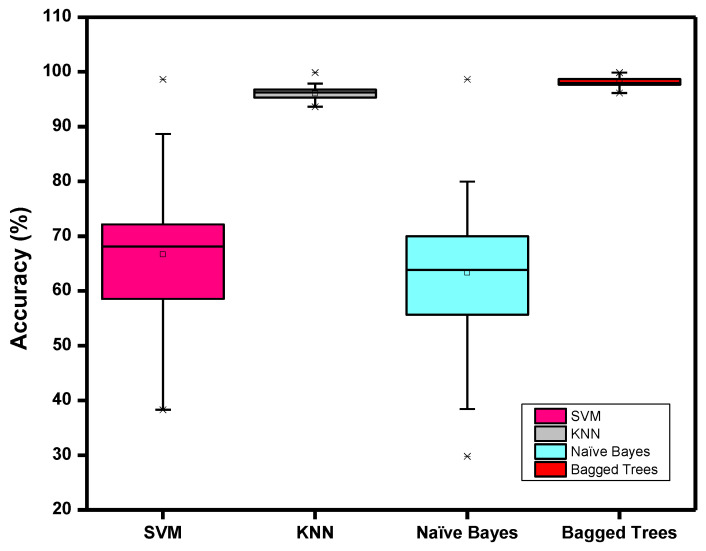
Comparison of the different machine learning algorithms.

We applied different machine learning algorithms to our dataset to find the best model in terms of accuracy, time complexity, and composability. We tested the support vector machine (SVM), K-nearest neighbor (KNN), naïve Bayes, and bagged trees models with each participant’s data. Figure 8 shows that the bagged trees model performed better than the other models in terms of accuracy and variance. The SVM and naïve Bayes models had an average accuracy of less than 70% with a larger interquartile range (IQR), suggesting a big difference in the performance of the models using the participant’s data. While the KNN and bagged trees models performed well with an overall accuracy of over 90%, we chose the bagged trees model, which showed less variance with the participant’s data indicated by the smaller IQR.

**Figure 9:**
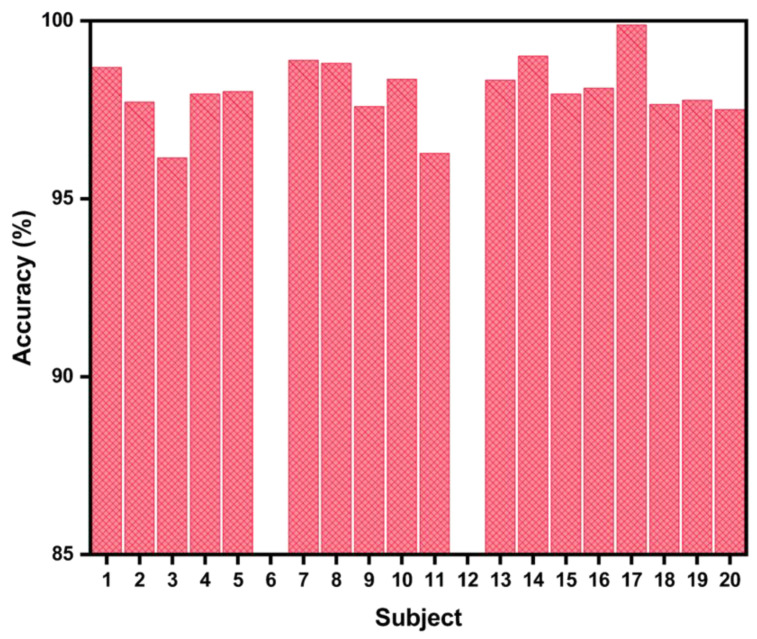
The accuracy of the individual TSV prediction model using the bagged trees algorithm.

Bagging is a machine learning procedure that can reduce the variance by making a subset and avoid overfitting by taking the average from each subset. This study used the bagged tree classification to classify individual thermal sensations. The dataset was divided into different combinations of training and testing ratios (60:40, 70:30, and 80:20) for each participant. The accuracy was increased by using 80% as the training set for each participant. Therefore, 80:20 was randomly selected as the training-to-testing set ratio, respectively. All analyses were performed using MATLAB (R2020b), and a 5-fold cross-validation was used during the training of the model. The accuracy of the individual prediction model was higher than 95% for all participants, as shown in Figure 9. The accuracy can be misleading for classification problems if the datasets are imbalanced. The experiments were performed on the subway train, and the temperatures inside the train were almost constant during the entire journey, and so were the TSVs of the participants. For this reason, the datasets were imbalanced, with the majority voting for a specific class. To evaluate the performance of each model, we also calculated the precision, recall, and *f*-1 score, as shown in Table 4, along with the accuracy using the following Equations (1)–(4):

(1) $$\mathrm{Accuracy}\;=\;\frac{\mathrm{TP}\;+\;\mathrm{TN}}{\mathrm{TP}\;+\;\mathrm{TN}\;+\;\mathrm{FP}\;+\;\mathrm{FN}}\;\times \;100\%$$

(2) $$\mathrm{Precision}\;=\;\frac{\mathrm{TP}}{\mathrm{TP}\;+\;\mathrm{FP}}\;\times \;100\%$$

(3) $$\mathrm{Recall}\;=\;\frac{\mathrm{TP}}{\mathrm{TP}\;+\;\mathrm{FN}}\;\times \;100\%$$

(4) $$f\;-\;1\;\mathrm{score}\;=\;\frac{2\;\times \;\mathrm{precision}\;\times \;\mathrm{recall}}{\mathrm{precision}\;+\;\mathrm{recall}}\;\times \;100\%$$

Since this was a multiclass problem, we took a one vs. all approach to calculate the metrics and finally measured the weighted average. August is hotter than other months in Seoul, and unfortunately, during this study, it was raining, so there were minimal temperature differences between the subway cabin and outside, which may have affected thermal sensation.

This study found that the SKT and EBT, along with their extracted features, can be used to predict individual thermal sensations. Skin is the largest organ of the human body and plays a vital role in thermoregulation by exchanging heat between the body and the ambient air. The laboratory testing in our previous experiment [40] found that EBT is significantly correlated with TSVs. Besides predicting individual thermal sensations in a subway, the researchers were trying to leverage the use of face masks by integrating sensors to monitor SKT, EBT, respiratory patterns, CO_2_ monitoring, biomarkers for inflammation, and airborne pathogen detection [57,58,59,60,61].

## 4. Conclusions

We designed a smart face mask to measure individual SKT and EBT and developed a smartphone application to store the data for analysis. We also proposed wavelet-based denoising and feature creation techniques, which showed a maximum accuracy of 100% with the bagged trees algorithm. The experiments were performed in real-time in a subway cabin under naturalistic experimental conditions. The SKT, EBT, and extracted features showed a significant correlation (*p* < 0.01), with an overall accuracy of 98.14% and an *f*-1 score of 96.33%. We plan to integrate ambient air temperatures and CO_2_ monitoring sensors, which will extend the usability of this smart face mask in future studies. All the participants in this study were young male university students. Further studies need to be done on different age groups and genders. Additionally, studies need to be conducted in other seasons to see the transient conditions between subway cabins and outside environments.

## Figures and Tables

**Table 1:** Details of the sensors used for this study.

Measurement	Sensor	Specification
Facial skin temperature	Infrared MLX90614-DCC	Operating voltage: 3.3–5 V, accuracy: ±0.2 °C, interface: I2C, response time: 0.15 s
Exhaled breath temperature	Thermistor NXFT15XH103FA2B130	Resistance at 25 °C: 10 KΩ, operating range: −40–125 °C, accuracy: ±0.8 °C, response time: 4 s

**Table 2:** Average age and physical information of the participants.

Number of Participants	Age (Years)	Height (cm)	Weight (kg)
20	23.1 ± 5	174.4 ± 5.5	77 ± 30

**Table 3:** The correlation of all the input features with participants’ TSVs.

ID	SKT	SKT_approx_	SKT_decomp_	EBT	EBT_approx_	EBT_decomp_
1	0.016	0.013	0.009	0.767 **	0.775 **	−0.011
2	0.472 **	0.473 **	0.003	0.133 **	0.131 **	0.002
3	0.118 **	0.117 **	0.054 **	0.231 **	0.216 **	0.021
4	0.302 **	0.304 **	−0.001	0.06 **	0.058 **	0.018
5	0.608 **	0.622 **	0.001	0.325 **	0.337 **	0.001
6	-	-	-	-	-	-
7	0.619 **	0.621 **	−0.003	0.4 **	0.414 **	0.004
8	0.012	0.007	0.008	0.078 **	0.075 **	0.007
9	−0.401 **	−0.402 **	−0.008	−0.097 **	−0.107 **	−0.001
10	0.249 **	0.247 **	0.002	0.252 **	0.253 **	−0.002
11	0.147 **	0.154 **	−0.019	−0.255 **	−0.264 **	−0.005
12	-	-	-	-	-	-
13	0.43 **	0.43 **	0.013	0.397 **	0.4 **	0.017
14	0.468 **	0.468 **	0.011	−0.068 **	−0.066 **	−0.006
15	0.022	0.021	−0.003	0.218 **	0.217 **	0.008
16	0.084 **	0.083 **	0.004	0.005	0.005	−0.001
17	−0.141 **	−0.141 **	0.009	0.04 **	0.042 **	−0.02
18	−0.217 **	−0.221 **	−0.003	0.29 **	0.29 **	−0.006
19	0.489 **	0.491 **	−0.004	0.223 **	0.226 **	0.005
20	0.348 **	0.348 **	−0.004	0.001	0.003	−0.001

** statistically significant (*p* < 0.01).

**Table 4:** Performance metrics of individual prediction model with the bagged trees algorithm.

ID	Precision (%)	Recall (%)	*f*-1 Score (%)	Accuracy (%)
1	97.40912879	97.405359	97.40108	98.69067
2	95.49638989	95.5277754	95.50511	97.7176
3	92.50089658	92.6493112	92.47093	96.14679
4	95.92654775	95.9369586	95.93012	97.94101
5	96.07387951	96.0685809	96.07052	98.01398
6	-	-	-	-
7	97.79543872	97.8439462	97.80826	98.8894
8	97.63298034	97.6421645	97.63427	98.80881
9	95.25275546	95.2461255	95.24822	97.59388
10	96.7478617	96.7516882	96.74953	98.35943
11	92.69322156	92.7050666	92.69831	96.27451
12	-	-	-	-
13	96.70350709	96.7725463	96.72947	98.33398
14	98.02587008	98.0287653	98.02712	99.00717
15	95.92848156	95.9575363	95.93169	97.93935
16	96.25907571	96.2621746	96.2548	98.10875
17	99.77315689	99.7731569	99.77316	99.88392
18	95.34922926	95.3577003	95.35017	97.64471
19	95.5944613	95.6120342	95.60206	97.76786
20	95.07726541	95.0968453	95.07749	97.50459

## Data Availability

Data underlying the results presented in this paper are not publicly available at this time but may be obtained from the authors upon reasonable request.

## Supplementary Materials

The following supporting information can be downloaded at: https://www.mdpi.com/article/10.3390/bios12121093/s1, Video S1: Connection with a smartphone after wearing the smart face mask; Video S2: Real-time acquisition of physiological and psychological data of a person wearing the smart face mask.

## Appendix A

**Table A1:** Average number of passengers entering and exiting the Gireum and Sadang station platforms of Seoul Metro in the morning, evening and during experiment [61].

Station		Morning	Evening	During Experiment
Gireum	Entering	2269	678	763
	Exiting	575	2050	1094
Sadang	Entering	2133	1081	914
	Exiting	831	1742	1046
